# Mitral prolapsing volume is associated with increased cardiac dimensions in patients with mitral annular disjunction

**DOI:** 10.1007/s12471-021-01575-6

**Published:** 2021-05-04

**Authors:** P. Luyten, S. Heuts, E. Cheriex, J. R. Olsthoorn, H. J. G. M. Crijns, B. Winkens, J. W. Roos-Hesselink, P. Sardari Nia, S. Schalla

**Affiliations:** 1 Department of Cardiology, Maastricht University Medical Centre, Maastricht, The Netherlands; 2 Department of Cardiology, Laurentius Hospital Roermond, Roermond, The Netherlands; 3 Department of Cardiothoracic Surgery, Maastricht University Medical Centre, Maastricht, The Netherlands; 4 Cardiovascular Research Institute Maastricht (CARIM), Maastricht University, Maastricht, The Netherlands; 5 Department of Methodology and Statistics, Faculty of Health, Medicine and Life Sciences, Maastricht University, Maastricht, The Netherlands; 6 Department of Cardiology, Erasmus University Medical Centre, Rotterdam, The Netherlands; 7 Department of Radiology and Nuclear Medicine, Maastricht University Medical Centre, Maastricht, The Netherlands

**Keywords:** Mitral regurgitation, Mitral annular disjunction, Prolapsing volume, Echocardiography

## Abstract

**Introduction:**

In patients with mitral annular disjunction (MAD), it can be difficult to assess the severity of mitral regurgitation (MR), as they present with a prolapsing volume (i.e. volume resulting from mitral valve prolapse, blood volume shift) rather than a regurgitant jet. The influence of the mitral prolapsing volume (MPV) on cardiac dimensions is unknown. We hypothesised that the severity of MR is underestimated in these patients. Our aim was to measure MPV and to investigate its influence on cardiac dimensions in patients with MAD.

**Methods:**

We retrospectively included 131 consecutive patients with MAD from our institution’s echocardiographic database. Transthoracic echocardiography was used to assess MPV. Additionally, we established a control group of 617 consecutive patients with degenerative mitral valve disease and performed propensity score matching.

**Results:**

Median MPV in the MAD group was 12 ml. MPV was an independent predictor for left ventricular end-diastolic (LVEDD) and end-systolic diameter (LVESD) and left atrial volume (all *p* < 0.001). In patients with large prolapsing volumes (> 15 ml), LVEDD (56 ± 6 mm vs 51 ± 6 mm, *p* < 0.001), LVESD [38 mm (34–41) vs 34 mm (31–39), *p* < 0.01] and left atrial volume [105 ml (86–159) vs 101 ml (66–123), *p* = 0.04] were significantly increased compared to matched patients with degenerative mitral valve disease and similarly assessed severity of MR.

**Conclusion:**

Due to a volume shift based on the MPV rather than an actual regurgitant jet, MR severity cannot be assessed adequately in MAD patients. Increased MPV induces ventricular and atrial enlargement. These findings warrant future studies to focus on MPV as an additional parameter for assessment of the severity of MR in MAD patients.

**Supplementary Information:**

The online version of this article (10.1007/s12471-021-01575-6) contains supplementary material, which is available to authorized users.

## What’s new?

- Severity assessment of mitral regurgitation (MR) can be difficult in patients with mitral annular disjunction (MAD).
- Patients with MAD present with a prolapsing volume rather than a regurgitant jet.
- Increased prolapsing volume was associated with an increase in cardiac dimensions.
- The prolapsing volume could be included in severity assessment of MR in MAD patients.

## Introduction

**Fig. 1:**
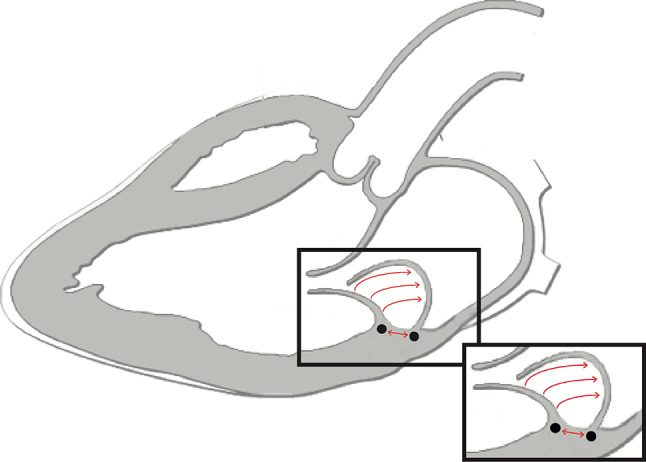
Schematic representation of mitral annular disjunction, showing displacement of the mitral hinge point, away from the ventricular myocardium towards the left atrium. The prolapse results in a volume shift without an effective regurgitant jet

During systole, the mitral valve annulus copes with strong forces, such as annular translation, contraction and folding [1]. In addition to annular dilatation and leaflet prolapse [2], Hutchins at al. reported that mitral annular disjunction (MAD) can be seen in patients with mitral regurgitation (MR) (especially in those with Barlow’s disease (BD)), which is defined as a separation between the atrial wall-mitral valve junction and the left ventricular muscle attachment (Fig. 1; [3]). They concluded that the floppy mitral valve develops from hypermobility of the valve apparatus, which is secondary to disjunction. Recent studies revealed that MAD is present in 42–55% of BD cases, predominantly in women [4, 5].

As it remains unknown what the effect of MAD might be on mitral valve prolapse and subsequent MR, it is important to explore the haemodynamic consequences of MAD. It is widely accepted that increasing effective regurgitant orifice area is a predictor of an unfavourable outcome in MR patients [6]. However, routine measurements to grade the severity of mitral valve regurgitation may not be applicable to MAD patients. Moreover, MAD patients with no or little regurgitation may have more severe regurgitation (i.e. volume shift) than initially assessed [7]. This volume shift can be observed as an additional volume displacement towards the left atrium (LA), meaning that an additional part of the left ventricular end-diastolic volume is not part of the stroke volume, potentially leading to an underestimation of the severity of MR according to current guidelines.

In addition to ventricular and atrial dilatation due to regurgitation, we hypothesise that mitral prolapsing volumes (MPVs) in mitral valves with MAD can also have an impact on left atrial and ventricular dimensions. As this volume shift is not part of the stroke volume, this could subsequently lead to volume overload, even if MR is not quantified as severe. As recently described, bulging of the floppy mitral valve into the LA could be measured by delineating the MPV by cardiac magnetic resonance imaging (CMR) [7], but in our view this can be done by echocardiography as well.

Furthermore, in most mitral pathologies, left atrial volume increases with increasing MR severity [8, 9]. To our knowledge, this has not been reported in MAD patients. We hypothesise that patients with MAD, independent of MR severity, still exhibit significant atrial and ventricular enlargement, compared with non-MAD patients.

The aim of the current study is to examine the effect of the MPV on the left atrial and ventricular dimensions in patients with MAD.

## Methods

In this observational single-centre retrospective analysis we identified in the Maastricht University Medical Centre echocardiography database all patients with MAD who underwent transthoracic echocardiography (TTE) between January 2009 and December 2015. Patients with MAD on TTE were identified by a senior imaging cardiologist with > 30 years’ experience (E.C.). MAD was defined as a clear separation between atrial wall-mitral valve junction and the left ventricular muscle attachment.

To acquire a control group consisting of patients with degenerative mitral valve regurgitation without MAD, we also evaluated the echocardiography database for all patients labelled as ‘degenerative mitral valve disease’ between 2009 and 2015.

Medical records were studied to retrieve biometric data, severity of dyspnoea based on the New York Heart Association (NYHA) scale, atrial fibrillation (AF) and coronary artery disease. If there was no history of AF, all electrocardiograms (ECGs) were reviewed to verify sinus rhythm. The presence of coronary artery disease was examined by means of the patient’s history, ECG review or analysis of any available coronary imaging. The need for informed consent was waived by our institutional review board, due to the observational character of the study.

Data were obtained using a Philips IE33 workstation (Philips Healthcare, Eindhoven, The Netherlands). Left ventricular end-diastolic diameter (LVEDD) was measured in the parasternal long axis view after closure of the mitral valve. Left ventricular end-systolic diameter (LVESD) was measured in the same view after closure of the aortic valve. Echocardiographic measurements were examined by two independent cardiologists (P.L., E.C.) and categorised according to the most recent guidelines [10, 11]. In cases of disagreement, images were reassessed by both cardiologists and a consensus was reached.

**Fig. 2:**
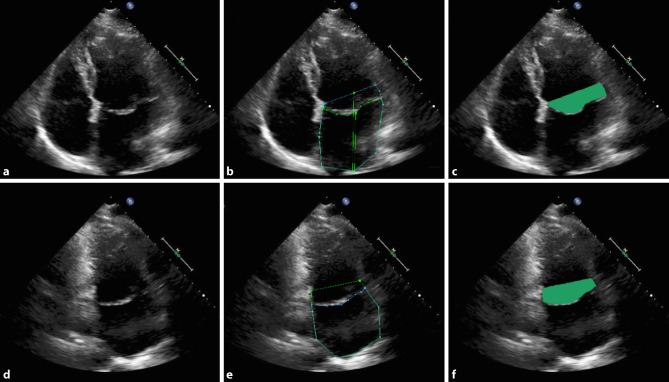
**a**–**f** Echocardiographic evaluation and quantification of mitral prolapsing volume. **a**–**c** Four-chamber view demonstrating **a** mitral annular disjunction, **b** measurements of atrial dimensions up to the level of the annulus (*blue*) and the mitral valve (*green*), resulting in **c** the prolapse surface. **d**–**f** Two-chamber view in the same patient demonstrating **d** mitral annular disjunction, **e** measurements and **f** prolapse surface

Left atrial volume was calculated by the modified Simpson’s method [12]. To define MPV, the LA was traced at maximum disjunction by the modified Simpson’s method using apical four- and two-chamber views (Fig. 2a, b). First, the LA was traced from the mitral annulus to the atrial border and then back to the annulus. Second, the LA was traced again from annulus to atrial border, but now above the prolapsing mitral valve. In order to determine the MPV the result of the first measurement was deducted from that of the second.

Categorical data are presented as numbers and percentages (%). Variables are presented as median (range) or mean ± standard deviation (SD) according to normality of distribution.

Univariable and multivariable regression was performed to identify predictors of either left atrial volume, LVESD and LVEDD.

Propensity score matching was performed to match the ‘disjunction group’ with the ‘degenerative group’, to assess differences in cardiac dimensions between these groups. Covariates included in the model were determined using the multivariable regression model. A caliper of 0.15 was used with nearest neighbour matching in a 1:1 ratio.

Statistical analyses were carried out using commercially available software (IBM SPSS Statistics for Windows, Version 25.0, Armonk, NY, USA); matching was performed in R through the SPSS R‑plugin package (PSMATCHING3 R Extension v3.03).

## Results

For the study group, 131 consecutive patients meeting the inclusion criteria for MAD were identified in our centre. Baseline characteristics of the 131 patients are summarised in Tab. 1. Mean age was 64 years and 49% of these patients were male. Approximately one-third of the patients had a history of AF and most patients were in NYHA functional class I and II for dyspnoea. Patients had a predominantly normal left ventricular ejection fraction (Tab. 1). Approximately one-third of the mitral valve regurgitation was classified as severe. Median left atrial volume was 103 ml (35–310) and median MPV was 12 ml (1–43).

**Table 1:** Characteristics of mitral annular disjunction patients

Baseline characteristics	*n* = 131
Age (years)	64 (17–90)
Gender (male)	64 (49%)
BSA (m^*2*^*)*	1.80 (1.67–1.95)
Atrial fibrillation	47 (36%)
Coronary artery disease	20 (15%)
Myocardial infarction	10 (8%)
*NYHA classification*	
I	50 (38%)
II	61 (47%)
III	20 (15%)
IV	0
LVEF%	62 (57–66)
LVESD (mm)	35 (31–39)
LVEDD (mm)	53 ± 7
TR jet velocity (m/s)	2.4 (2.1–2.8)
*MR gradation*	
Mild	49 (37%)
Moderate	34 (26%)
Severe	48 (37%)
*Left atrial volume (ml)*	104 (77–143)
*Mitral prolapsing volume (ml)*	12 (7–17)

Data are number and percentage (%), median (range) or mean ± standard deviation

*BSA* body surface area, *NYHA* New York Heart Association, *LVEF* left ventricular ejection fraction, *LVESD* left ventricular end-systolic diameter, *LVEDD* left ventricular end-diastolic diameter, *TR* tricuspid regurgitation, *MR* mitral regurgitation

In univariable regression analysis, age, gender, body surface area (BSA), MPV and MR severity were associated with LVEDD. These factors were assessed in a multivariable model, in which a significant regression equation was found [F(5.118) = 11.498, *p* < 0.001] with an *R*^2^ of 0.328. In this model, BSA, MPV and MR severity were independently associated with LVEDD (Tab. 2).

**Table 2:** Univariable and multivariable regression analysis for left ventricular end-diastolic diameter (*LVEDD*), left ventricular end-systolic diameter (*LVESD*) and left atrial volume

Variable	*R*	*p*-value	B	β	CI	*p*-value
*Correlation LVEDD*	*Univariable*		*Multivariable*			
Age	0.21	0.02	−0.04	−0.10	−0.09–0.02	0.23
BSA	0.52	< 0.01	9.86	0.34	4.07–15.64	< 0.01
Gender	0.34	< 0.01	−0.96	−0.08	−3.27–1.36	0.42
Mitral prolapsing volume	0.38	< 0.01	0.06	0.18	0.01–0.26	0.04
MR gradation	0.23	0.01	0.57	0.23	0.61–2.88	< 0.01
Atrial fibrillation	0.11	0.22				
Coronary artery disease	0.10	0.24				
*Correlation LVESD*	*Univariable*		*Multivariable*			
Age	0.22	0.12	−0.25	−0.08	−0.08–0.03	0.37
BSA	0.41	< 0.01	6.45	0.25	0.87–12.01	0.02
Gender	0.30	< 0.01	−0.76	−0.07	−2.99–1.50	0.50
Mitral prolapsing volume	0.35	< 0.01	0.135	0.20	0.01–0.26	0.03
MR gradation	0.09	0.31				
*Correlation left atrial volume*	*Univariable*		*Multivariable*			
Age	0.09	0.32				
BSA	0.38	< 0.01	54.42	0.25	18.73–90.12	< 0.01
Gender	0.25	< 0.01	−0.95	−0.10	−15.74–13.85	0.90
Mitral prolapsing volume	0.29	< 0.01	0.86	0.16	0.05–1.67	0.04
MR gradation	0.45	< 0.01	23.42	0.43	16.14–30.70	< 0.01
Atrial fibrillation	0.44	< 0.01	−31.20	−0.32	−44.22–−18.17	< 0.01
Coronary artery disease	0.06	0.51				

*BSA* body surface area, *CI* confidence interval, *MR* mitral regurgitation

For LVESD, univariable regression analysis found age, gender, BSA and MPV to be associated. A multivariable model found a significant regression equation [F(4.119) = 7.598, *p* < 0.001] with an *R*^2^ of 0.203. In this model, BSA and MPV were associated with LVESD (Tab. 2).

For left atrial volume, gender, BSA, MPV and AF were predictive. The subsequent model then found a significant equation [F(4.119) = 13.171, *p* < 0.001] with an *R*^2^ of 0.484. In this model, BSA, MPV, MR severity and AF proved to be independent predictors of left atrial volume (Tab. 2). Thus, BSA and MPV were the only parameters associated with all three parameters: LVEDD, LVESD and left atrial volume.

For the degenerative mitral valve disease control group, 617 consecutive patients were identified and included for analysis. Pre-matching baseline characteristics are presented in the Electronic Supplementary Material (Table S1). As found in the multivariable models, covariates such as age, gender, BSA and MR severity were included in the matching model. Eventually, 105 pairs were matched. Characteristics of these pairs are presented in the Electronic Supplementary Material (Table S2). After matching, there were no persisting differences in baseline characteristics.

While the whole range of prolapsing volumes was included in these analyses (median 11 ml, range 1–43 ml, interquartile range 7–17 ml), we assessed the influence of a large prolapsing volume (> 15 ml, LPV). Tab. 3 shows the differences between the two groups (LPV vs degenerative group) for symptomatology and echocardiographic findings. We found gender to differ significantly between the groups (71% males vs 50% males, *p* = 0.041). No further differences in demographics were observed. The LPV group exhibited significantly larger left atrial volumes [105 ml (86–159 ml) vs 101 ml (66–123 mL), *p* = 0.040], larger LVEDD (56 ± 6 vs 51 ± 6, *p* < 0.001) and larger LVESD [38 mm (34–41 mm) vs 34 (31–39 mm), *p* = 0.005].

**Table 3:** Characteristics for prolapsing volumes > 15 ml

	Large prolapsing volume (> 15 ml) *n* = 31	Degenerative group *n* = 105	*p*-value
Age (years)	62 (47–75)	67 (54–77)	0.204
Gender	22 (71%)	52 (50%)	0.041
BSA	1.9 (1.8–2.1)	1.9 (1.7–2.0)	0.179
Coronary artery disease	2 (7%)	14 (13%)	0.525
Atrial fibrillation	11 (36%)	38 (36%)	1.000
*NYHA*			0.302
I	12 (38%)	50 (48%)	
II	9 (29%)	42 (40%)	
III	10 (33%)	13 (12%)	
*MR severity*			0.640
Mild	12 (38%)	46 (44%)	
Moderate	9 (29%)	22 (21%)	
Severe	10 (33%)	37 (35%)	
Mitral prolapsing volume	22 (17–26)	0	
Left atrial volume	105 (86–159)	101 (66–123)	0.040
LVEDD	56 ± 6	51 ± 6	< 0.001
LVESD	38 (34–41)	34 (31–39)	< 0.01
LVEF	62 (57–65)	62 (55–65)	0.913

Data are number and percentage (%), median (range) or mean ± standard deviation

*BSA* body surface area, *NYHA* New York Heart Association classification for dyspnoea, *MR* mitral regurgitation, *LVEDD* left ventricular end-diastolic diameter, *LVESD* left ventricular end-systolic diameter, *LVEF* left ventricular ejection fraction

## Discussion

In our MAD patient cohort, we observed a blood volume shift towards the LA due to bulging of the valve into the LA, subsequent to disjunction. However, this finding has not yet been described or quantified, and its haemodynamic consequences and effect on MR severity have not been studied.

Severity assessment of MR is based on qualitative, semi-quantitative and quantitative measurements [10, 13]. Notably, only one semi-quantitative measurement (pulmonary vein flow) takes a volume shift towards the LA into consideration [14].

We observed a volume shift towards the LA due to MAD, which could hypothetically lead to an underestimation of MR severity. Therefore, the aim of the current study was to examine the effect of MPV on the left atrial and ventricular dimensions in MAD patients.

The main finding of the current study is that the MPV due to MAD is associated with increased left ventricular and atrial dimensions. Furthermore, MPV is not related to the conventionally assessed MR severity, meaning that the current MR severity assessment is insufficient to determine the influence of MPV on atrial and ventricular dimensions (which is due to retrograde volume displacement rather than to a regurgitation jet in MAD patients).

Based on our findings, we propose novel terminology to assess MR severity in MAD patients. MR itself is a regurgitant jet, flowing through the mitral valve back into the LA. In contrast, the MPV in MAD patients is volume displacement, not part of the stroke volume, while still contained within the mitral valve.

If we regard MR as a volume, then the MPV would also be part of this regurgitant volume. However, MR is mostly calculated by the proximal isovelocity surface area (PISA) method. In MAD patients, this would not be valid because the volume is contained within the valve [15].

This also explains the difference in severity assessment of MR between echocardiography and CMR. CMR measures differences in stroke volume by planimetry of left and right ventricular volumes and flow measurements of the ascending aorta [7, 16]. This means that severity assessment in MAD patients would be more accurate using CMR rather than echocardiography following current echocardiographic guidelines.

Although we did not find significant differences regarding cardiac dimensions in the overall matched groups, we sought to evaluate the potential influence of LPV (> 15 ml) on cardiac dimensions. We found significantly larger LVEDD, LVESD and left atrial volume in the LPV group. There was a significant difference in gender distribution between these groups. However, this was not considered important as multivariable regression analyses for these dimensions revealed gender not to be an independent predictor of any of the cardiac dimensions. This is probably due to the independent predictive value of BSA (which is gender-dependent) for these dimensions [17], which is corrected for in a multivariable model. There were no differences in any other baseline characteristics. These findings suggest that small prolapsing volumes (< 15 ml) do not influence cardiac dimensions, in contrast to larger volumes (> 15 ml).

Although we did not find a difference in the incidence of AF between the groups, it is generally accepted that patients with larger left atrial volumes are more prone to develop AF [18, 19] while, on the other hand, AF begets increased left atrial volumes [20].

Finally, we did not find a relationship between MR severity and MPV, although we did find larger cardiac dimensions in patients with LPV. However, MPV is not in any way used as a parameter in current severity assessment, while MPV does independently negatively influence cardiac dimensions, and potentially also symptomatology. Subsequently, these findings imply that the MR severity of these patients could be underestimated using the current severity assessment standards.

Our study has several limitations. First, this study is retrospective in nature. We have limited potential bias by using propensity score matching to correct for known covariates and baseline differences. However, AF was not taken into consideration for propensity score matching analysis. Still, this important predictor for left atrial volume did not differ between the two groups.

Second, by formation of a new group (LPV) these potential biases were reintroduced, although there were no important differences in baseline characteristics between the studied groups. Finally, the regurgitant volume (including MPV) could potentially be measured more accurately with CMR, although echocardiography is the most widely used technique.

Therefore, the above-mentioned limitations could encourage future studies to use at least a combination of these modalities to assess MR severity in this specific patient group.

## Conclusion

Mitral annular disjunction represents a special entity in mitral valve disease. Patients with MAD may present with various degrees of MR, which is difficult to assess accurately with current echocardiographic parameters.

In the present study, we describe a new parameter in patients with MAD that has an effect on cardiac dimensions and function: mitral prolapsing volume. Patients with prolapsing volumes > 15 ml present with larger ventricular and atrial dimensions. This finding implies that the severity of MR in these patients could be underestimated using the current standard of severity assessment. Future prospective studies are warranted to determine the exact influence of various prolapsing volumes on ventricular dimensions and clinical outcomes.

## Supplementary Information

*Supplementary Data S1*. Pre-matching demographic characteristics. *Supplementary Data S2*. Post-matching demographic and echocardiographic characteristics.
