# Supplementary material for: Mitral prolapsing volume is associated with increased cardiac dimensions in patients with mitral annular disjunction
Source: Neth Heart J. 2021 May 4;30(3):131–9. doi: 10.1007/s12471-021-01575-6 (PMC8881558; doi:10.1007/s12471-021-01575-6)
Supplement: Supplementary file 1 — Supplementary Data S1. Pre-matching demographic characteristics. Supplementary Data S2. Post-matching demographic and echocardiographic characteristics. [file 12471_2021_1575_MOESM1_ESM.docx]

Supplementary Data S1. Pre-matching demographic characteristics

|  | Disjunction group  n=131 | Degenerative group  n=617 |
| --- | --- | --- |
| *Age* | 64 [49-76] | 68 [57=77] |
| *Gender (male)* | 64 (49%) | 352 (57%) |
| *BSA* | 1.8 [1,7-2,0] | 1.8 [1,7-2,0] |
| *MR severity* |  |  |
| *Mild* | 49 (37%) | 242 (39%) |
| *Moderate* | 34 (26%) | 265 (43%) |
| *Severe* | 48 (37%) | 110 (18%) |

*BSA: Body Surface Area, MR: Mitral Regurgitation*

Supplementary Data S2. Post-matching demographic and echocardiographic characteristics

|  | Disjunction group  n=105 | Degenerative group  n=105 | p-value |
| --- | --- | --- | --- |
| *Age (years)* | 64 [53-77] | 67 [54-77] | 0.722 |
| *Gender* | 55 (52%) | 52 (50%) | 0.783 |
| *BSA* | 1,8 [1,7-2,0] | 1,9 [1,7-2,0] | 0.133 |
| *Coronary artery disease* | 17 (16%) | 14 (13%) | 0.698 |
| *Atrial fibrillation* | 39 (37%) | 38 (36%) | 1.000 |
| *NYHA* |  |  | 0.239 |
| *I* | 38 (36%) | 50 (48%) |  |
| *II* | 50 (48%) | 42 (40%) |  |
| *III* | 17 (16%) | 13 (12%) |  |
| *MR severity* |  |  | 0.530 |
| *Mild* | 42 (40%) | 46 (44%) |  |
| *Moderate* | 29 (28%) | 22 (21%) |  |
| *Severe* | 34 (32%) | 37 (35%) |  |
| *Mitral prolapsing volume* | 11 [7-17] | 0 |  |
| *LA volume* | 101 [76-141] | 101 [66-123] | 0.188 |
| *LVEDD* | 52 ± 6 | 51 ± 6 | 0.279 |
| *LVESD* | 35 [31-38] | 34 [31-39] | 0.900 |
| *LVEF* | 62 [58-66] | 62 [55-65] | 0.246 |

*BSA: Body Surface Area, NYHA: New York Heart Association classification for dyspnea, MR: Mitral Regurgitation, LA: Left atrium, LVEDD: Left Ventricular End Diastolic Diameter, LVESD: Left Ventricular End Systolic Diameter, LVEF: Left Ventricular Ejection Fraction.*
